# Perceptions of sugar mommy practices in South Africa

**DOI:** 10.1080/14330237.2014.906086

**Published:** 2014-10-28

**Authors:** Nancy Phaswana-Mafuya, Olive Shisana, Adlai Davids, Cily Tabane, Margaret Mbelle, Gladys Matseke, Mercy Banyini, Queen Kekana

**Affiliations:** ^a^ Human Sciences Research Council, South Africa; ^b^ Office of the Deputy Vice Chancellor: Research, Technology, Nelson Mandela Metropolitan University, Port Elizabeth, South Africa; ^c^ University of the Witwatersrand, Johannesburg, South Africa; ^d^ University of Venda, Thohoyandou, South Africa

**Keywords:** intergenerational, older women, sexual transactions, South Africa, sugar mommies, younger men

## Abstract

The study sought to explore sugar mommy practices regarding their occurrence, acceptability as well as perceived reasons why older women and younger men enter into sugar mommy relationships. An exploratory qualitative study involving 135 participants from 11 diverse focus groups in terms of age, gender (females=27%) and geotype throughout the nine South African provinces was conducted. Data on the participants’ views, opinions and experiences of sugar mommy practices were collected using focus group interviews. The data were thematically analyzed. The study found that sugar mommy practices were prevalent in South Africa. The perceived reasons for acceptability were: love, survival, and correctness. Perceived reasons why older women have sexual relationships with younger men included: sexual fulfilment, domination, reduction of stress, physical attraction, procreation, lack of self-control, youthful feeling, migrancy, difficulty in finding partners of compatible age and young men being seen as not demanding. Perceived reasons why younger men have sexual relationships with older women included: material gain, reduction of stress, being enticed, rejection by women of compatible age, peer influence and belief that older women are purer. Given the increase in sugar mommy practices, which may have significant implications for the prevalence of HIV/AIDS, it is necessary to understand the underlying perceptions of these practices, in order to develop culturally relevant and socially acceptable intervention programmes.

## Introduction

“Age mixing”, “cross-generational sex” and “intergenerational sex” are terms which have been used interchangeably to refer to sexual relationships between sexual partners whose age gap is 10 years or more (Feldman- Jacobs & Worley, 2008; Parker & Hajiyiannis, 2008). These terms have mainly been portrayed as involving sexual relationships between younger females and older men, commonly referred to as sugar daddies. A review of “more than 45 qualitative and quantitative studies in Sub-Saharan Africa revealed that sugar daddy practices were common and were associated with unsafe sexual behaviour” (Luke, 2003, p.67). In particular, studies found that sugar daddy practices increased the risk of HIV infection as these sexual relationships were often characterized by low condom use, among other factors (Glynn, Carael, Auvert, Kahindo, Chege, Musonda, Kaona, & Buvé, 2001; Gregson, Nyamukapa, Garnett, Manson, Zhuwhu, & Carael et al., 2002; Kelly, Gray, Sewankambo, Serwadda, Wabwire-Mangen, Lutalo, & Wawer, 2003; Langeni, 2007; Shisana, Rehle, Simbayi, Zuma, Jooste, Pillay-van-Wyk, Mbelle, Van Zyl, Parker,Zungu, Pezi, & the SABSSM Implementation Team, 2009) A related phenomenon of sexual relationships between older women (commonly referred to as sugar mommies) and younger men is reported to be on the increase (Forsloff, 2009). Lawrence (2003) found that in the USA, almost 33% of women between 40 and 60 years were dating men aged 10 or more years younger than themselves. While relationships between older women and younger men have been cited in several studies in sub-Saharan Africa, most of them do not provide much detail about the reasons that underlie these practices nor the level of HIV risk that these relationships have. A study conducted in South Africa in 2005 (Shisana, Rehle, Simbayi, Parker, Zuma, Bhana, Connolly, Jooste, & Pillay, 2005), revealed that approximately 34% of women over 40 years of age were dating younger men. Sugar mommy practices have also been identified as occurring in other countries. For example, sugar mommy practices are considered as one of the contextual factors for increased HIV risk in Gaborone, Botswana (Tabane, 2004) and Yaounde, Cameroon (Meekers & Calves, 1997). One study in Zimbabwe reported that about two-thirds of the boys had had at least one sexual experience with a woman at least 10 years older (Chinake, Dunbar, van der Straten, Esim, Makunike, Vere, & Padian, 2002). In Uganda, the sugar mommy phenomenon also seems commonplace given that the practice is featured even in popular magazines (Gysels, Pool, & Nyanzi, 2005).

### Influences of sugar mommy relationships

Most studies (though limited) only report the occurrence of sugar mommy practices and there remains a need to understand what encourages or sustains them. Given the increase in sugar mommy practices, it is necessary to understand the underlying perceptions of these practices, in order to develop culturally relevant and socially acceptable intervention programmes. Sugar mommy practices may increase the risk of contracting HIV dramatically. In South Africa, Shisana et al. (2009) found higher HIV prevalence rates among women between 25 and 29 years of age compared to the rest of women in the other age categories. The study further revealed that males under 20 years of age had a relatively lower HIV prevalence compared to older males in the late twenties and early thirties (ibid). As in the case of older men (sugar daddies) having sex with younger women and girls, inter-generational sex between older women and younger men and boys also demands attention, especially given the differing prevalence of HIV between these groups. Maundeni (2004) asserts that: “*It is difficult for boys to practice safer sex with older women; this is because the women are often in control of the relationship, as they have more resources than boys”* (p.50). This power dynamic may increase HIV risk among younger men who may not exercise safer practices for fear of losing the sugar mommy. On the other hand, younger men who are involved in sexual relations with sugar mommies may concurrently have regular younger sexual partners, thus increasing exposure of HIV risk to women in the younger age group. The study sought to understand sugar mommy practices in South Africa. The following questions guided the research:

a) Do sugar mommy practices happen in your community?
b) Are they acceptable or unacceptable?
c) Why do you think individuals enter into these sorts of relationships?

## Method

### Participants and setting

In total, eleven (11) diverse focus group discussions (FGDs) comprising 135 participants participated in the study across nine provinces (see Table 1 below). An average of 10 participants participated in each FGD. Participants were selected purposively to participate in the study.

**Table 1.** Summary of study participants

### Procedure and data collection

Permission and approval for the study was granted by the HSRC's Research Ethics Committee (REC 2/23/10/07). Participants were accessed through various social networks including community organizations or leaders. The participants individually consented to participate in the study. Data were collected by trained research assistants.

The guiding questions that were prepared and contained in the fieldworkers’ manual were as follows:

a) Do sugar mommy practices happen in your community?
b) Are they acceptable or unacceptable?
c) Why do you think individuals enter into these sorts of relationships?

The FGDs were tape recorded and transcribed *verbatim* into text formats. During the discussion, the co-facilitator also kept session notes in order to check the accuracy of the transcripts. The session notes were also used for checking the accuracy of transcripts with participants. Some light refreshments were served during the FGDs as recommended in social research and in keeping with the philosophy of *ubuntu* (humanism) which is widely practiced in South Africa. Each FGD took an average of 60 minutes.

### Data handling

The moderating researcher and the researcher who took notes and operated the tape tallied consent forms, read through the notes and clarified statements resulting from poor sound quality, whilst the information was still fresh in their minds. Tapes were sent out to professionals for translation and transcription. Once these were completed, they were sent to the HSRC researchers who inserted notes in relevant sections of the transcripts to clarify the context in which these statements were made. Transcripts were centrally handled by two researchers who ensured that all focus group transcripts from six provinces had been submitted. Summaries and tables were developed for ease of identifying each transcript. Transcripts were circulated by e-mail to the authors for qualitative analysis.

### Data analysis

Data were thematically analyzed using thematic content analysis. Thematic content analysis steps involved: going through all eleven FGD transcripts; identifying recurring themes or topics and sub-themes; looking for underlying meanings, similarities and differences between them and grouping them together and naming them (Miles & Huberman, 1994). Researchers worked together in the same room in order to engage on possible interpretations, meanings and also to arrive at the most accurate interpretation of quotations. Researchers paid attention to the meanings of the words and phrases expressed by participants and their context based on participants’ own vocabu-laries (Miles & Huberman, 1994).

## Results

The results are presented according to the three FGD guiding questions, namely: occurrence of sugar mommy practices in communities, acceptability or unacceptability of sugar mommy practices and perceived reasons why individuals engage in sugar mommy practices (see Table 1). Attribution of statements is to focus group location, gender and sexual orientation. Table 2 lists the themes and subthemes that emerged from the study and will be elaborated on below.

**Table 2.** Themes and subthemes of the study

### Occurrence of sugar mommy practices

Participants across provinces and groups indicated that sugar mommy practices do happen in their communities. Participants were of the view that the majority of sugar mommies were resourceful women who hold senior posts, especially in government. They were generally perceived as one's right and choice.

*I think most of the time it is caused and encouraged by women. If you take a closer look most women who occupy high government posts are the ones who form part of a bigger statistic. They resort to younger men as they give lot of money to them. (*Males, Mokopane, Limpopo)

*I know a guy who was doing matric and he was in love with our female teacher. They were not hiding their relationship. This teacher used to give this guy a car; they would kiss and everybody will see. (*Witbank FGD)

*Yes it does happen, who cares?* (Women who have sex with women (WSW), Atteridgeville)

*Yeah, common things*. ***(***WSW, Western Cape)

In few instances (from rural locations), participants indicated that sugar mommy practices did not happen in their communities as this was regarded as an insult and a health hazard.

*It differs across communities, in our community it is not practiced; it is regarded as an insult but we can say love is not determined by age but it is not used in our community. (*Adult males, Limpopo)

*People still think that when a young man sleeps with an older woman the chances are that the young man might develop a kidney condition and some think that he might not bear children or impregnate a woman as he grows (older) and therefore shy away from sugar mommy practices*. (WSW, Atteridgeville)

* … in my community, it is not common; it is regarded as an insult … * (Adult male, Limpopo)

*Or maybe is in the suburbs; not here in the community*. (WSW, Western Cape

*No, it's not as much as like before*. (Male teenagers, Northern Cape)

### Acceptability and non-acceptability of sugar mommy practices

Some participants viewed sugar mommy practices as acceptable. They commented about love, survival, and correctness.

#### Love knows no age limit

Typical responses were:

* … love doesn't ask for age … * (Northern Cape, male teenagers)

*Time changes and age does not count anymore*. (WSW, Gauteng)

*Age is only a number and nothing else*. (North West, adult females)

*The right age is a myth*. (Free State, mixed teenagers)

*People should have space to choose what they want*. (Free State, mixed teenagers)

#### It is a means of survival, social or economic gain

*Some will accept it because if the older person now gives him money to make love to her, then the child will bring the money home so that the parents can buy food or electricity or so*. (Northern Cape, male teenagers)

* … other parents are happy because their standard of living will be improved*. (North West, adult females)

*Some other wives accept such behaviour to let their husbands go for women who have more money. Some even open the accounts to save this money. So they even don't worry even when their husbands spend 3 days outside with other women as long as they come back with money*. (Males of all ages, Limpopo)

 … *I have finished my studies and I am looking for a job; then I meet this lady, and she loves me. She then supports me financially and I use her money to support my family as well because they took me through school. I can now manage to maintain them though I do not have a job. So parents are not against the whole affair because this lady gives me money. Yeah, the greatest thing above all is because they like money, not that they like the whole thing* (Male teenagers, Eastern Cape)

#### There is nothing wrong with it

The view that was expressed was that if older men could have sexual relationships with younger women, then older women should have relationships with younger men.

*I personally see nothing wrong with that*. (Free State, mixed teenagers)

### Perceived reasons for non-acceptability of sugar mommy practices

The perceived reasons for non-acceptability of sugar mommy practices among males of different age groups across provinces were: the relationship is driven by lust, financial gain and inappropriate social behaviour.

#### The relationship is driven by lust

* … when older people try to advise young men, they say they want to test what their parents tested (sexual desire) so adults discontinue to advise them. (*Adult males, Limpopo)

#### The relationship is driven by financial gain

*They are interested in money … what other reasons are there that a young man will go out with an older woman?* (Male teenagers, Northern Cape)

* … young men use drugs before they go to these women to kill their conscience because they do it to get money because they know that these relationships are not accepted*. (Adult males, Limpopo)

* … young men will lack a good sense of responsibility because they are always receiving and they give less in return*. (WSW, Gauteng)

#### Inappropriate social behaviour

*I think what causes this is lack of ethical and moral values. If you are an older woman and have a relationship with men younger than your age this shows that you have a serious problem in promoting ubuntu … * (Adult male, Limpopo)

* … this is an indication of lack of self-respect … * (Males of all ages, Limpopo Province)

### Perceived reasons why older women have sexual relationships with younger men

The perceived reasons why older women have sexual relationships with younger men, across age groups, gender and geographic location, included: sexual fulfilment, domination, stress relief, physical attraction, procreation, lack of self-control, youthful feeling, migrancy, not finding partners of compatible age and young men seen as not demanding.

#### Sexual fulfilment

The main reason given within and across groups as to why older women have sexual relationships with younger men was to fulfil sexual needs that spouses or sex partners of their own age cannot fulfil. The reasons advanced were that older men do not provide sexual pleasure and satisfaction due to ill-health, low sexual drive, physical inactivity, poor sexual performance and loss of sexual interest, while on the contrary younger men provide better sex, are highly sexually active, strong, healthy, fresh, gentle and energetic. Participants indicated that men normally lose interest much earlier than women and this in turn forces women to hunt for younger blood in order to satisfy their sexual cravings.

Funa igazi lo mfana*, (want the blood of the young boy). If the man at home does not service well at home, so the woman goes and looks for a young boy because younger men give better sex; they still have the energy, they are therefore highly sexually active and give older women all the satisfaction they need*. (Male teenagers, Northern Cape)

*Older men suffer from different diseases like sugar diabetes and high blood pressure which affect their sexual performance*. (Adult men, Limpopo; mixed male and female teenagers, Free State)

#### Domination

Participants within and across groups indicated that sugar mommies would like to have someone they can instruct to do what they want them to do. Older women use their resources to exert some influence or domination over the younger sex partner. Younger men find themselves having no choice but to oblige lest they lose all the benefits they are getting from the relationship such as clothes and pocket money.

*The woman wants the man to do what she wants him to do … the woman wants to sit on his head. The man does things the woman wants him to do. Maybe it's sometimes like – what do they call it now – black … blackmail. The sugar son has no room to refuse; it is more of instructing them than requesting them to do that. The sugar mommy will instruct the younger man to drive around to do groceries, to the hair salon or to go pick her children from school and the sugar son does not have room enough to refuse. It is more of instructing them than requesting them to do that*. (Male teenagers, Northern Cape)

#### Reduction of Stress

Participants within and across groups cited reduction of stress as one of the reasons why sugar mommies have sexual relationships with younger men. Sugar mommies may be experiencing work or family related stress, or be in loveless marriages where they are cheated on or being physically and emotionally abused by their spouses. To free themselves from all these worries and take revenge on their spouses, they indulge in sex with younger men.

*Sometimes the cause of all this could be a situation a woman finds herself in, … you'll find a man cheating and the woman start behaving like that too or the women wanting to be loved because their husbands are beating them and undermine them or their men left them for younger girls*. (Mixed adult men and women, Mpumalanga)

#### Physical attraction

Physical attraction and fitness of the younger man were perceived as some of the motives why older women have sex with younger men.

*The muscles are maybe nicer there*. (Male teenagers, Northern Cape)

*As men grow older, they get out of shape; they get big belly and they look older than [their] women*. (Adult women, North West)

#### Procreation

Several male teenagers in the Northern Cape FGD mentioned that sugar mommies have these relationships in order to get pregnant.

*For a baby … to want a baby; maybe she's never had a child … now … she really wants one. She knows that she is not going to want to adopt someone else's child, or so – now she feels, ‘no, okay, I'm then just going to fall pregnant’*. (Male teenagers, Northern Cape)

#### Lack of self-control

One group mentioned that lack of self control in the older women, especially when under the influence of alcohol, was a cause for sugar mommy practices.

*In the shebeen, sometimes these older women want these younger boys to buy liquor for them and later in the evening they invite them to accompany them to their houses. Sometimes they help themselves (urinate) not far from these young men and this makes them [younger man] to have sex with them; some older women do not respect themselves to these young men*. (Adult men, Limpopo)

#### Youthful feeling

Some participants from two groups cited wanting to have a youthful feeling as a reason for older women wanting to have relationships with young men.

*They believe that having sex with younger men keeps them young. They say they feel young and loved again because when you are in a marriage for a long time you grow apart and there is no more love but only tolerance. Younger men call them with all the romantic loving words and they feel loved again. In such relationships they think the young guys listen to them and follow the instruction unlike when they are in a relationship with someone of their age*. (WSW, Gauteng)

#### Migrancy

One of the reasons cited as contributing to sugar mommy practices was the distance between partners due to migratory labour. Participants were of the view that when husbands or regular sex partners are working away from home, young men are regarded as better sex replacements than partners of their own age.

*Sometimes it is caused by when the man of the family is working far from his family, for example, working in Gauteng [province] and [his] wife, feeling shy to go for men of her age then decides to go for younger men*. (Adult men, Limpopo)

#### Difficulty in finding partners of compatible age

One of the reasons why women have sex with younger men was cited as the difficulty in finding men of compatible age or men of compatible age not being interested or available.

*You find that the woman is no longer fit to have relationships with men of her age because they reject her, so she tends to go for young men for sex, she finds that she doesn't have a choice*. (Adult males, Limpopo)

#### Young men seen as not demanding

Participants indicated that older women have sex with younger men because they are easy and appreciate most things when compared to older men.

*Older women are not as demanding as older men. Young men are satisfied by anything you give him*. (WSW, Gauteng)

### Perceived reasons why younger men have sexual relationships with older women

The reasons why younger men have sexual relationships with older women included: material gain, reduction of stress, being enticed, rejection by women of compatible age, peer influence and belief that older women are purer. Some of these have been reported on in previous section.

#### Material gain

As previously noted, the younger men had sexual relationships with older women for material gain in order to fulfil their personal and family needs as well as interests. Younger men may be selective or target potential “service providers”. Sugar mommies spoil their sugar sons by buying them fashionable clothes, giving them allowances, buying them cell phones and even cars, if they can.

*Sugar mommies are called ATM's. So … it's about money – fancy car, jewellery, expensive clothes, big cell phone, big wallet, lots of cards, credit cards, debit cards, everything! Even though … dating the older woman, he still has a lot of girlfriends and uses the older woman's BMW to attract them*. (Males of all ages, Limpopo)

*Hmmm, they also have cars, stay in town houses and younger men are still at school. On pay day these young men receive an incentive from sugar mommies … * (WSW, Western Cape)

*She used to give me money. I slept with her once I ran away thereafter*. (Mixed adult men and women, Mpumalanga)

#### Reduction of stress

Participants indicated that younger men sometimes think that their age group girls cause them too much stress, moreso than older people. Therefore, getting involved with an older person gives them less or no stress. They were of the opinion that older women are more mature, more respected, have the ability to deal with life's challenges and are more experienced in sex and life in general.

* … the treatment that you get from the sugar mommies and also the way that she, she treats you, treating you well, more than the same age; she also pampers him and makes him feel loved*. (Mixed teenagers, Limpopo)

#### Being enticed

Participants indicated that at times younger men may have sexual relationships with older women because of being enticed through the wearing of revealing clothes or inviting them to an environment which is conducive to sex.

* … sometimes these women call young men to fix something like radio in their houses; when they arrive they find the woman wearing a towel. The woman makes it a point that the towel falls leading them to have sex instead of fixing the radio as agreed*. (Males of all ages, Limpopo)

*The teacher wears a very short skirt and while in front of the class, she writes on the board and her body is petite especially when she is bending. So maybe hey, … The teacher is killing me. After that I make sure that I meet with the teacher, and … sometimes she had aimed for that too*. (Males, Eastern Cape)

*When you see an older woman dressed in a mini skirt and walking in the public like that, it remains obvious that she is trying to invite younger men by saying I'm still young and beautiful*. (Males, Limpopo)

#### Rejection by women of compatible age

*Young men find themselves being rejected by women of their ages so this results in them loving older women*. (Males of all ages, Limpopo)

#### Peer influence

Participants indicated that young men were influenced by their friends with regard to material benefits from relationships with older women. They go for sugar mommies so that, like their friends, they can get resources which will make them superior among their peers.

*I support the statement that says “birds of the same colours fly together; so once your friends do something you tend to do the same thing*. (Males of all ages, Limpopo)

*Some younger men are interested in loving older women because they have seen their peers having more money from older women. So they tend to join the other peers as they regard it as good life in order to have money*. (Males of all ages, Limpopo)

#### Belief that older women are purer

Participants indicated that some young men went for older women because they believed that they were clean; they have no sexually transmitted diseases because they do not have concurrent sexual partners and they have resources to look after their health.

*There is an ongoing belief among younger men which says older women won't make you sick they are as clean as a baby. You won't go wrong with older women*. (Males, Limpopo)

## Discussion

This study revealed that sugar mommy practices do exist in South African communities. This type of relationship may be on the increase internationally (Maundeni, 2004). The practice of older women having sexual relationships with younger men and even marrying them is becoming normalized in contemporary society (Mstywrl, 2006). While sugar mommy practices may be successful in certain ways, they also pose personal-health-social risks for those involved in an era of HIV/AIDS. The sexual-social environment plays an important role in people's behaviour; people tend to behave as expected by those around them.

The main reasons for indulgence in sugar mommy practices as reflected in the findings are sexual fulfilment and financial gain. The literature (Varga, 1997; Varga, 2003; Wellings, Collumbien, Slaymaker, Singh, Hodges, Patel, & Bajos, 2006) shows that older partners are typically more empowered economically and the benefits of such relationships are viewed primarily as being financial (Leshnoff, 2008). Maundeni (2004) states that some of the younger boys engaged in relationships with older women because of a belief that older women make better lovers, older women are in possession of money of their own, the unavailability or unwillingness of the boys’ female peers and the feeling that female peers are immature. These findings are supported in other studies which found that young men have sexual relationships with older women because of the older women's emotional and sexual maturity which make them better equipped to teach younger men about life, love and sexuality (Borysenko, 2012) and to teach younger men more sensual pleasures (Mybrotha, 2009). However, younger men may be looking for mother figures, companionship – someone good to talk to and spend time with, a female (Leshnoff, 2008).

The study found that engagement in consensual and non-consensual sexual relationships between younger men and “sugar mommies” is usually influenced by a myriad of age-dependent motives such as domination, stress reduction, favours, and material benefits (Kuate-Defo, 2004). Whereas “sugar daddies” may be motivated by physical pleasure, “sugar mommies” may also seek emotional or other support in addition to being motivated by physical pleasure (Frank, Esterhuizen, Jinabhai, Sullivan, & Taylor, 2008). states that older women are not only interested in the stamina or “re-boot” of ability of the younger male, they also like the adventure of their more spontaneous, younger companion. He further mentions that women may also want a man with a less-developed career who could follow her (domination). These days women are choosing younger men just like men took off after younger women for a fling, admiration and pleasure. They are having fun without entanglement or embarrassment, and some of them make the temporary fling a permanent arrangement (Forsloff, 2009).

## Conclusion

Sugar mommy practices are becoming a more and more common and acceptable phenomena in South Africa. Perceived reasons why older women have sexual relationships with younger men included: sexual fulfilment, domination, reduction of stress, physical attraction, procreation, lack of self-control, youthful feeling, migrancy, difficulty in finding partners of compatible age and young men seen as being less demanding. Perceived reasons why younger men have sexual relationships with older women included: material gain, reduction of stress, being enticed, rejection by women of compatible age, peer influence and the belief that older women are purer. Given that the increase in sugar mommy practices may have significant implications for the spread of HIV/AIDS, it is necessary to understand the underlying perceptions of these practices, in order to develop culturally relevant and socially acceptable intervention programmes.
